# *mef2ca* and *mef2cb* Double Mutant Zebrafish Show Altered Craniofacial Phenotype and Motor Behaviour

**DOI:** 10.3390/biom13050805

**Published:** 2023-05-09

**Authors:** Andreia Adrião, Sara Mariano, José Mariano, Paulo J. Gavaia, M. Leonor Cancela, Marta Vitorino, Natércia Conceição

**Affiliations:** 1 Centre of Marine Sciences (CCMAR), Universidade do Algarve, Campus de Gambelas, 8005-139 Faro, Portugal; 2 Faculty of Sciences and Technology, Universidade do Algarve, Campus de Gambelas, 8005-139 Faro, Portugal; 3 Center of Physics and Engineering of Advanced Materials (CeFEMA), IST, Universidade de Lisboa, Av. Rovisco Pais, 1096-001 Lisboa, Portugal; 4 Faculty of Medicine and Biomedical Sciences, Universidade do Algarve, Campus de Gambelas, 8005-139 Faro, Portugal; 5 Algarve Biomedical Center, Universidade do Algarve, Campus de Gambelas, 8005-139 Faro, Portugal

**Keywords:** Myocyte enhancer factor 2 (MEF2C), autosomal dominant mental retardation syndrome-20 disease (MRD20), MEF2C haploinsufficiency syndrome, zebrafish (*Danio rerio)*, craniofacial development, *Mef2c* mutants

## Abstract

The transcription factor MEF2C is crucial in neuronal, cardiac, bone and cartilage molecular processes, as well as for craniofacial development. MEF2C was associated with the human disease MRD20, whose patients show abnormal neuronal and craniofacial development. Zebrafish *mef2ca*;*mef2cb* double mutants were analysed for abnormalities in craniofacial and behaviour development through phenotypic analysis. Quantitative PCR was performed to investigate the expression levels of neuronal marker genes in mutant larvae. The motor behaviour was analysed by the swimming activity of 6 dpf larvae. We found that *mef2ca*;*mef2cb* double mutants display several abnormal phenotypes during early development, including those already described in zebrafish carrying mutations in each paralog, but also (i) a severe craniofacial phenotype (comprising both cartilaginous and dermal bone structures), (ii) developmental arrest due to the disruption of cardiac oedema and (iii) clear alterations in behaviour. We demonstrate that the defects observed in zebrafish *mef2ca*;*mef2cb* double mutants are similar to those previously described in MEF2C-null mice and MRD20 patients, confirming the usefulness of these mutant lines as a model for studies concerning MRD20 disease, the identification of new therapeutic targets and screening for possible rescue strategies.

## 1. Introduction

Myocyte enhancer factor 2 (MEF2C) is a transcription factor present as a single gene in the human genome, located in chromosome 5 at position q14.3. MEF2C haploinsufficiency causes abnormal neuronal and craniofacial development leading to autosomal dominant mental retardation disease 20 (MRD20) or *MEF2C* haploinsufficiency syndrome [1].

*Mef2c* conditional inactivation in mouse cranial neural crest (CNC) cells led to multiple craniofacial malformations, delayed ossification and hypoplasia or loss of most of the skeletal elements of the face and skull. These results confirmed the crucial role of MEF2C in the CNC for proper craniofacial development, where it was found to function as a transcriptional activator and partner of distal-less homeobox 5 (Dlx5) and distal-less homeobox 6 (Dlx6) [2]. It was also found to be a direct target of endothelin signalling in the developing neural crest [3]. MEF2C is required for mice’s appropriate growth and development of endochondral bones. It was also shown that MEF2C regulates the hypertrophic differentiation of chondrocytes in endochondral bones and is a direct regulator of Collagen type X alpha 1 (*ColXa1*) transcription during chondrocyte hypertrophy [4].

Zebrafish is an excellent biomedical model with comparable physiological processes with humans [5]. Zebrafish has two *mef2c* genes, *mef2ca* and *mef2cb*, located in chromosomes 10 and 5, respectively, as determined by *in silico* analysis [6], and mutant lines are available for both genes [7,8]. The mutant line *mef2ca^b1086^* carries a mutation that leads to an early stop codon upstream of the MEF2 domain, likely corresponding to a loss-of-function allele. This mutant line shows a severe craniofacial phenotype, presenting inappropriate overdevelopment of skeletal structures [7]. It also shows an altered expression of several endothelin 1 (*edn1*)-dependent target genes, namely heart and neural crest genes derivatives expressed 2 (*hand2*), several distal-less homeobox genes (*dlx3b*, *dlx4b*, *dlx5a* and *dlx6a*), bagpipe homeobox homolog 1 (*bapx1*) and goosecoid (*gsc*), demonstrating that zebrafish Mef2ca acts downstream of Edn1 signalling in CNC cells [7], as also observed in mice [2,3].

The mutant line available for the *mef2cb* gene, *mef2cb^fh288^*, carries a mutation that leads to a premature stop codon in the MADS domain. These mutants show normal development with no disturbance of craniofacial or heart structures. However, larvae of the *mef2ca^b1086^*;*mef2cb^fh288^* double mutants corresponding to both Mef2ca and Mef2cb loss of function lack the majority of differentiated cardiomyocytes showing an abnormal heart formation and consequent developmental arrest. These results led to the conclusion that both MEF2C orthologues are essential for heart formation in zebrafish and that Mef2ca and Mef2cb function as redundant co-orthologues and are essential to drive cardiomyocyte differentiation [8]. In craniofacial development, larvae homozygous for *mef2ca^b1086^* mutation and heterozygous for *mef2cb^fh288^* show a phenotype with altered cartilaginous structures, revealing a partially redundant function of Mef2ca and Mef2cb in cartilage development [9].

The proteins methyl CpG binding protein 2 (MECP2) and cyclin-dependent kinase-like 5 (CDKL5) are responsible for normal brain development in high vertebrates, their dysfunction being related to human pathologies [10,11]. MEF2C was found to be a transcriptional regulator of those genes, and accordingly, patients with MEF2C haploinsufficiency syndrome showed a decreased expression of *MECP2* and *CDKL5* [12]. Zebrafish *mecp2* was shown to be expressed in embryonic and adult zebrafish brain tissues [13], and *mecp2* mutants showed an altered behavioural phenotype, similar to what occurs in humans with mutations in this gene [14]. Recently, we reported that *cdkl5* spatial-temporal expression in zebrafish is in agreement with its known localization in humans [15], and zebrafish *cdkl5* mutants also displayed an altered behaviour [16].

The objective of our work was to characterize the role of both MEF2C orthologues in zebrafish development through the usage of zebrafish mutants for both *mef2ca* and *mef2cb* genes. We described the resultant craniofacial phenotype and the motor activity in embryos and young larvae and analysed the expression of marker genes involved in neuronal development. We also developed a transgenic mutant line that allows the in vivo tracking of the development of the cartilaginous craniofacial structures and their forming abnormalities.

## 2. Material and Methods

### 2.1. Zebrafish Lines and Maintenance

All wild-type (WT), mutant and transgenic zebrafish lines were maintained in standard conditions in a recirculating system (ZebTec housing system; Tecniplast, Kettering, UK), with water at 28.5 ± 0.5 °C and a 14 h light/10 h dark photoperiod. System water was artificially produced and exchanged at a 10% daily rate, maintained with a conductivity of 760 ± 50 µS and pH of 7.8 ± 0.1. Fish were fed twice a day with dry food (Zebrafeed, Sparos, Olhão, Portugal) and live *Artemia nauplii.* Mutant line *mef2ca^b1086^* [7] was acquired from Zebrafish International Resource Center (ZIRC); *mef2cb^fh288^* [8] was kindly provided by Y. Hinits, King’s College London, United Kingdom; *Tg*(-4725*sox10*:GFP) [17] was kindly provided by R. Kelsh, Bath University, United Kingdom. Further crossing originated the double-mutant *mef2ca^b1086^*;*mef2cb^fh288^* and reporter mutant lines *mef2ca^b1086^*;*Tg*(-4725*sox10*:GFP) and *mef2ca^b1086^*;*mef2cb^fh288^*;*Tg*(-4725*sox10*:GFP). Genotyping was performed as previously described for *mef2ca^b1086^* and *mef2cb^fh288^* [8] using genomic DNA obtained from fin clips or whole zebrafish embryos. Briefly, sequencing of PCR products amplified using primers 5′-AAAGCAGGCAAATAGAAAAACACT-3′ and 5′-AAAAGGCCAAACTCAACAGGAACT-3′ for *mef2ca^b1086^* and 5′-GGAAGAAGCGCTGTATTTAGGAC-3′ and 5′-ATATCTGTGCTGGCGTACTGG-3′ for *mef2cb^fh288^*. All experiments were performed in compliance with European Union Directive 2010/63/EU/.

### 2.2. Tissue Labelling and Imaging

The alcian blue 8GX (Sigma-Aldrich, St. Louis, MO, USA) staining method for cartilage was adapted from Walker and Kimmel (2007) [18] and performed in 4% paraformaldehyde (pH 7.4 with PBS) fixed larvae to characterize craniofacial cartilaginous structures. The terminology used followed the study by Cubbage and Mabee (1996) [19]. Vital staining with 0.003% alizarin red S (Sigma-Aldrich) was performed as previously described [20], to characterize craniofacial calcified structures. Skeletal preparations were imaged on a SteREO Lumar V12 stereomicroscope (Carl Zeiss, Jena, Germany). Fluorescence images of skeletal structures, live fish imaging and live movies were made on an Olympus IX81 inverted microscope.

### 2.3. RNA Extraction and Quantitative Real-Time Polymerase Chain Reaction (qPCR)

Total RNA was prepared as previously described [21]. Samples were obtained from four different pools of 20 larvae with three days post fertilization (dpf) and sharing the same genotype: WT, *mef2ca^b1086/b1086^*, *mef2cb^fh288/fh288^* or *mef2ca^b1086/b1086^*;*mef2cb^fh288/fh288^*. RNA purification was performed with a High pure RNA isolation kit (Roche Diagnostics, Indianapolis, IN, USA) according to the manufacturer’s protocol and quantified by spectrometry (NanoDrop ND-1000; Thermo Fisher Scientific, Waltham, MA, USA). cDNA was prepared from 500 ng of DNase (Promega, Madison, WI, USA) treated total RNA from each sample by reverse transcription, using Moloney murine leukaemia virus (M-MLV) reverse transcriptase (Invitrogen, Life technologies, Carlsbad, CA, USA).

qPCR was performed in a CFX96 PCR detection system (Bio-Rad, Hercules, CA, USA). PCR reactions were prepared with 1× SsoFastTM EvaGreen Supermix (Bio-Rad), 0.2 µM of each primer (Table 1) and 2 µL of a 1/10 cDNA dilution. Reactions were submitted to an initial denaturation of 30 s at 95 °C followed by 40 amplification cycles (each cycle was 5 s at 95 °C, 10 s at 65 °C). For each assay, a negative control was performed in the absence of a cDNA template. Fluorescence was measured at the end of each extension cycle in the FAM-490 channel, and melting profiles of each reaction were performed to check for unspecific product amplification. Levels of gene expression were calculated using the relative quantification method 2^−ΔΔCt^ [22] and normalized using gene expression levels of *glyceraldehyde 3 phosphate dehydrogenase* (*gapdh*) as reference gene.

### 2.4. Analysis of Motor Behaviour

#### 2.4.1. Early Spontaneous Motor Behaviour

Experiments were performed on WT, *mef2ca^b1086/b1086^*, *mef2ca^b1086/+^*, *mef2cb^f h288/f h288^* and *mef2cb^f h288/+^* zebrafish lines with 25 h post fertilization (hpf), 51 hpf embryos and 6 days post fertilization (dpf) larvae. These three time-point tests were performed in the same larvae that were maintained individualized until the end of the experiments. Larvae were kept in 96-well plates (Nunclon Delta Surface, Thermo Fisher Scientific), filled with 250 μL of embryo medium, until they reached 48 hpf and then were transferred into 12-well plates (Nunclon Delta Surface, Thermo Fisher Scientific) filled with 2 mL of embryo medium.

Approximately 200 embryos with 24 hpf, per genotype, were mechanically dechorionated at least one hour before the experiments, using sharp forceps. Embryos were then acclimatized to room temperature for at least 20 min before the beginning of the experiments. To estimate the rate of spontaneous contractions, each freely moving embryo was recorded under a stereo-microscope (Leica MZ6, Wetzlar, Germany) using a digital camera (Canon PowerShot G12, Canon, Tokyo, Japan) attached to the microscope ocular, over a period of 2 min. Videos were later reviewed, and contractions were counted. Only complete coilings were counted. These were defined as the period between the first deviation of the trunk from its resting state until its return to its initial position. In the case of multiple contractions after a spontaneous movement, only the first coiling was considered.

#### 2.4.2. Spontaneous Swimming Activity

For the spontaneous swimming activity calculations, a maximum of 10 larvae per replicate and a maximum of 30 larvae per genotype with 6 dpf (26 WT, 29 *mef2ca^b1086/b1086^*, 19 *mef2ca^b1086/+^*, 30 *mef2cb^fh288/fh288^* and 30 *mef2cb^fh288/+^* larvae) were maintained in 12-well plates and used for normalization. Recordings were performed under a stereo-microscope using a digital camera, over a period of 3 min, at 24 frames per second, after a period of 20 min acclimatization to room temperature. The videos were processed using Tracker 4.96 software (copyright © 2016 Douglas Brown—physlets.org/tracker) to follow the movement. Coordinate axes were locked in the centre of the well, and a software virtual calibration stick was used to indicate the diameter of the well (17 mm). The track control created was a “point mass”. The function “Autotracker” was used, and all settings were set to default (“template evolution rate”—“20%”, “automark”—“4”, “search”—“look ahead”; the tail size was set to “none”).

For an object (one fish) occupying *N* positions *P_0_*, *P_1_*, …, *P_n_* over time, the tracking software produces a file with the coordinates and times of those positions:

(*x*_0_, *y*_0_, *t*_0_), (*x*_1_, *y*_1_, *t*_1_), …, (*x_n_*, *y_n_*, *t_n_*)

**Figure 1:**
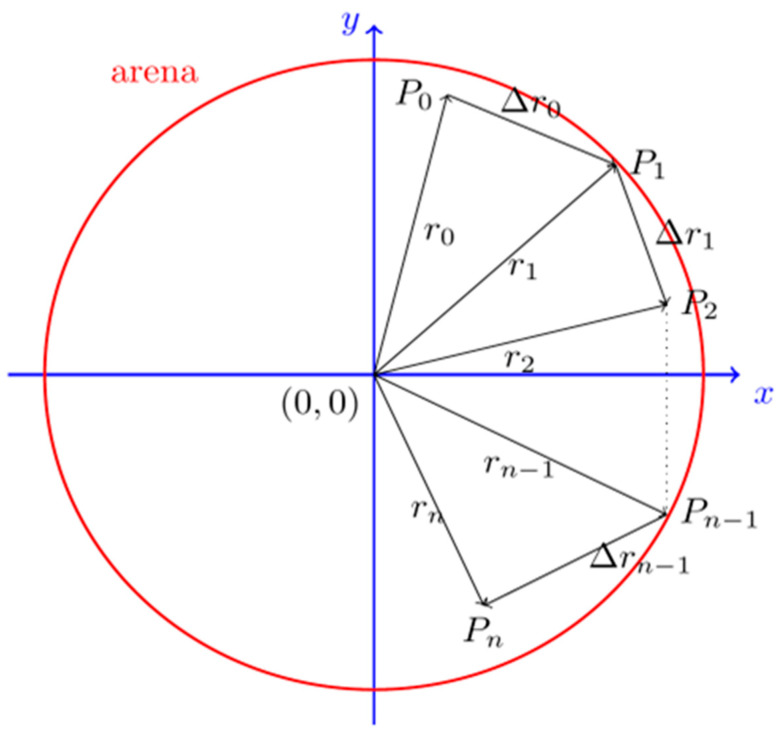
Successive positions of objects over time referred to the frame of reference used by the tracker software. The coordinate axis is represented by blue lines. The red circle represents the arena of radius 8.5 mm.

Refer to the coordinate system of the axis (Figure 1).

Following the same general lines proposed by Pietri et al. (2013) [14] to analyse motor behaviour, a set of metrics was defined, such as the total distance covered by each fish, the average of accelerations and the maximum of the average velocities. To compute those metrics, custom R scripts (http://www.R-project.org/ (accessed on 20 March 2017)) were developed.

Given a set of coordinates over time (*x_n_*, *y_n_*, *t_n_*), we calculated the following:

Radial distance (*r_i_*): the distance to the centre of the arena at time *t_i_* is given by the length of the line segment which joins the origin (0,0) with point (*x_i_*, *y_i_*):

$$r_{i}=\sqrt{x_{i}^{2}+y_{i}^{2}}$$

Distance covered (Δ*r_i_*): The  displacement between points *P_i_* and *P_i+_*_1_ is given by

$$\Delta r_{i}=\sqrt{{\left(x_{i+1}-x_{i}\right)}^{2}+{\left(y_{i+1}-y_{i}\right)}^{2}}$$

The total distance, *d*, travelled by each fish is the sum of the partial displacement Δ*r_i_*:

$$d=\overset{N-1}{\underset{i=0}{\sum }}\Delta r_{i}$$

Velocity (*v_i_*) between instants *t_i_* and *t_i+_*_1_ is given by

$$vi=\frac{\sqrt{{\left(x_{i+1}-x_{i}\right)}^{2}+{\left(y_{i+1}-y_{i}\right)}^{2}}}{\left(t_{i+1}-t_{i}\right)}=\frac{\Delta r_{i}}{\Delta t_{i}}$$

The average of velocities is given by

$$\bar{v}=\frac{1}{N-1}\overset{N-1}{\underset{i=0}{\sum }}v_{i}$$

Acceleration (*a_i_*) is given by

$$a_{i}=\frac{\left(v_{i+1}-v_{i}\right)}{\left(t_{i+1}-t_{i}\right)}=\frac{\Delta v_{i}}{\Delta t_{i}}$$

The average of accelerations is given by

$$\bar{a}=\frac{1}{N-2}\overset{N-2}{\underset{i=0}{\sum }}a_{i}$$

### 2.5. Statistical Analysis

All results were expressed as means ± standard deviation (SD) of measurements from at least three independent experiments. One-way analysis of variance (ANOVA) using Tukey’s multiple comparison test was performed to make comparisons between two groups using Graph Pad Prism 6.0 (Graph Pad software, Boston, MA, USA). Differences were considered statistically significant for *p* < 0.05.

For the zebrafish locomotor behaviour, the data were analysed using R software (R core team), and the results were plotted using R and Microsoft Excel. Results are presented using histograms, bar graphs with confidence intervals and box-and-whisker diagrams with the median as the central red mark, the first quartile (q1) and third quartile (q3) for the edge of the box (black) and the extreme data points represented by the whiskers in the range of q1 − 1.5 × (q3 − q1) to q3 + 1.5 × (q3 − q1).

Since the distribution of the data was unknown, statistical significance was assessed using the Kruskal–Wallis test [23].

## 3. Results

### 3.1. Disruption of Cardiac Oedema Causes the Death of Homozygous Double-Mutant Larvae

**Figure 2:**
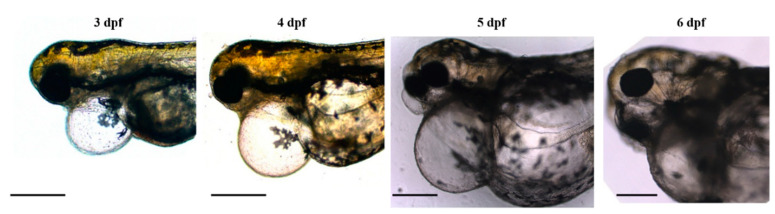
Phenotype evolution of double homozygous larvae (*mef2ca^b1086/b1086^*;*mef2cb^fh288/fh288^*) from 3 to 6 dpf of development. The images were obtained at different times of development showing an increase in cardiac oedema, with a consequent general deformation that leads to complete immobilization of the animal. Scale bars: 600 µm.

Zebrafish homozygous double-mutant larvae for both *mef2c* co-orthologues (*mef2ca^b1086/b1086^*;*mef2cb^fh288/fh288^*) were obtained through the crossing of the mutant lines available, *mef2ca^b1086^* and *mef2cb^fh288^*. The evolution of the overall phenotype due to Mef2ca and Mef2cb loss of function in larvae development was analysed by direct imaging throughout the development of several single larvae. Images from homozygous double-mutant larvae with developmental stages ranging from 3 to 6 dpf are shown in Figure 2. We observed the development of cardiac oedema that resulted in the swelling of the anterior portion of the body and deformation of the specimen with consequent immobilization. The cardiac disruption occurred at 6 dpf and led to fish death.

### 3.2. Homozygous Double Mutants Show a Severe Craniofacial Phenotype with Alterations in Cartilaginous and Bone Structures

**Figure 3:**
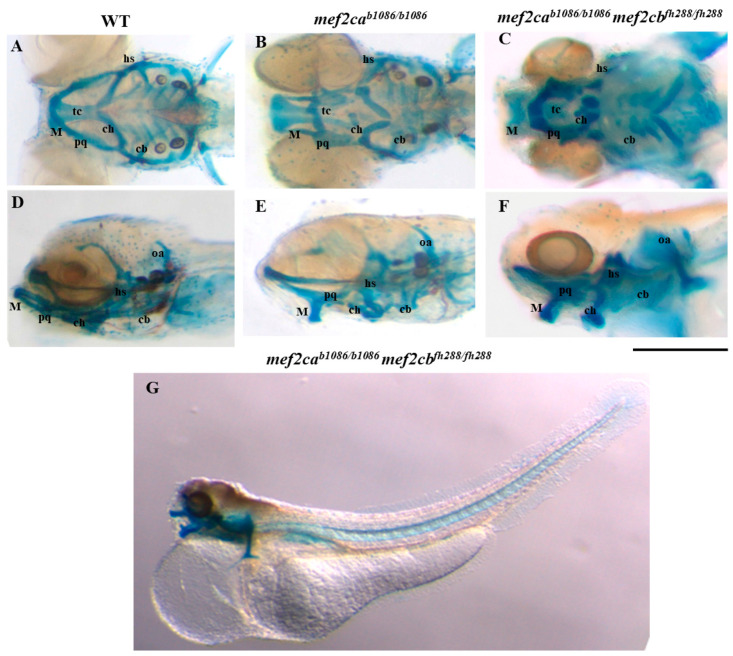
Craniofacial cartilage phenotypes in WT, *mef2ca* single-mutant and homozygous double-mutant larvae. The alcian blue staining technique was used to analyse craniofacial cartilaginous phenotype in zebrafish larvae at 5 dpf. (**A**–**C**) Ventral view of WT, *mef2ca* single-mutant or homozygous double-mutant larvae. (**D**–**F**) Lateral view of WT, *mef2ca* single-mutant or homozygous double-mutant larvae. (**G**) Lateral view of the whole body of homozygous double-mutant larvae. hs—hyosymplectic cartilage; M—Meckel’s cartilage; ch—ceratohyal; pq—palatoquadrate cartilage; cb—ceratobranchials; oa—occipital arch; tc—trabecula communis. Scale bars: 600 µm.

Zebrafish homozygous double-mutant larvae for both *mef2c* co-orthologues showed a severe cartilage phenotype as revealed by alcian blue staining, consisting in a severe disarrangement of the craniofacial structures when compared to *mef2ca* single-mutant (*mef2ca^b1086/b1086^*) and WT larvae at the same developmental stages (Figure 3). All the cartilaginous structures, such as branchial arches and ceratohyal, basihyal, hyosymplectic, palatoquadrate and Meckel’s cartilage showed a reduced size. The otic capsules are formed in a different position. Ceratohyals were displaced ventrally and fused with basihyals. Meckel’s cartilage was displaced ventrally originating a non-functional mouth.

**Figure 4:**
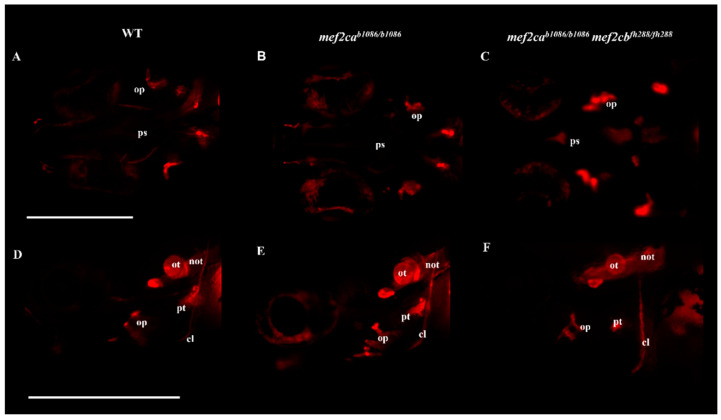
Craniofacial bone phenotypes in WT, *mef2ca* single mutants and homozygous double-mutant larvae. The alizarin red S live staining technique was used to analyse craniofacial bone phenotype in zebrafish larvae at 6 dpf. (**A**–**C**) Ventral view of WT, *mef2ca* single-mutant and homozygous double-mutant larvae. (**D**–**F**) Lateral view of WT, *mef2ca* single-mutant and homozygous double-mutant larvae. op—operculum; cl—cleithrum; ps—parasphenoid; ot—otoliths; pt—pharyngeal teeth; not—notochord. Scale bars: 600 µm.

Analysis of the craniofacial bone structures was performed using a live staining technique with alizarin red S. Homozygous double-mutant larvae showed alterations both in the structure and levels of mineralization, when compared to WT and *mef2ca* single mutants, at the same stage of development. The opercula and ethmoid plate were found to have an altered shape, with a smaller size and showing a higher level of mineralization. Otoliths were displaced, and the basioccipital process showed an altered morphology. The pharyngeal teeth had a reduced size and were displaced (Figure 4).

**Figure 5:**
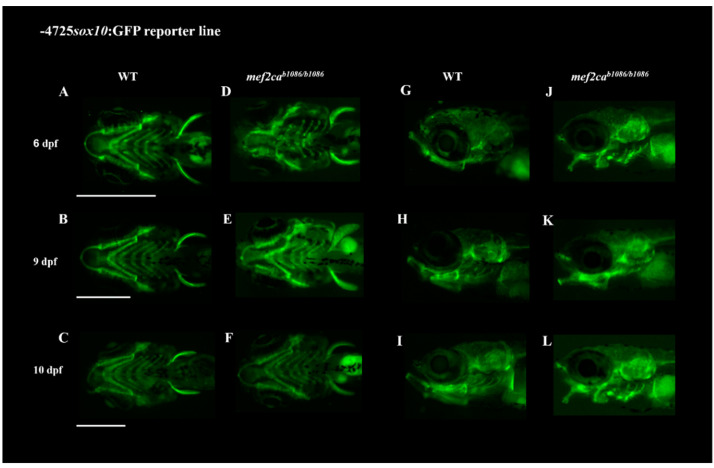
Craniofacial phenotype of *mef2ca* single mutants through the use of the transgenic line Tg(*-4725sox10:GFP*). Crossing the transgenic line Tg(*-4725sox10:GFP*) with the zebrafish *mef2ca^b1086^* mutant line allowed the observation in vivo of the craniofacial phenotype from these mutants. (**A**–**C**,**G**–**I**) WT larvae at developmental stages of 6, 9 and 10 dpf in ventral and lateral views, respectively. (**D**–**F**,**J**–**L**) *mef2ca^b1086/b1086^* homozygous larvae at developmental stages of 6, 9 and 10 dpf in ventral and lateral views, respectively. Scale bars: 600 µm.

**Figure 6:**
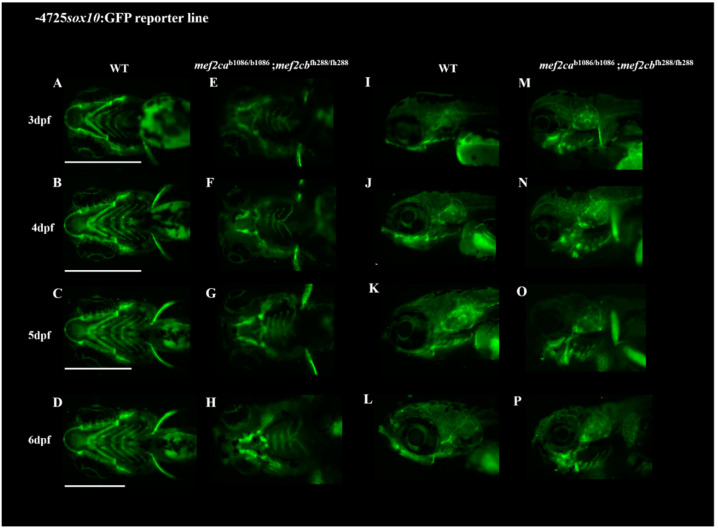
Craniofacial phenotype of double homozygous mutants through the use of the transgenic line Tg(*-4725sox10:GFP*). Crossing the transgenic line Tg(*-4725sox10:GFP*) with the zebrafish *mef2ca^b1086^;mef2cb^fh288^* mutant line allowed the observation in vivo of the craniofacial phenotype from these mutants. (**A**–**D**,**I**–**L**) WT larvae at developmental stages ranging from 3 to 6 dpf, in ventral and lateral views, respectively. (**E**–**H**,**M**–**P**) *mef2ca^b1086/b1086^;mef2cb^fh288/fh288^* double homozygous larvae at developmental stages from 3 to 6 dpf, in ventral and lateral views, respectively. Scale bars: 600 µm.

Crosses between *mef2ca* single- and double-mutant lines with the transgenic line Tg(*-4725sox10:GFP*) resulted in reporter mutant lines that allow in vivo observation of the cartilaginous craniofacial phenotype of these mutants. Transgenic *mef2ca* single-mutant and homozygous double-mutant larvae were followed until 10 dpf and 6 dpf, respectively (Figure 5 and Figure 6). All the cartilaginous craniofacial structures were easily observed in vivo under fluorescence microscopy, and the increment of the craniofacial cartilaginous disarrangement from homozygous *mef2ca* to double mutant, previously observed with the alcian blue staining method, was easily visualized in these reporter transgenic lines, validating and confirming their usefulness in following in vivo the development of the mutant craniofacial phenotypes.

### 3.3. Zebrafish mef2c Mutants Showed Alteration of mecp2 and cdkl5 Gene Expression

**Figure 7:**
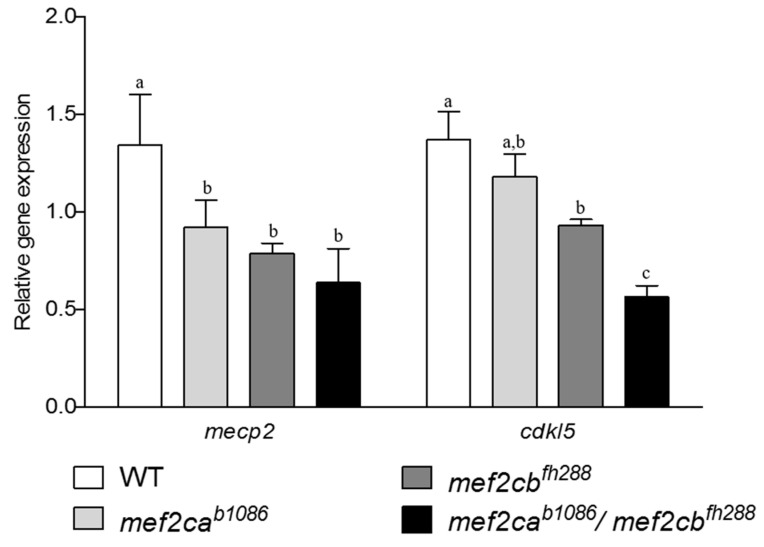
Expression analysis of brain-development-related genes, *mecp2* and *cdkl5,* in zebrafish *mef2ca* and *mef2cb* single mutants and homozygous double mutants. Gene expression was measured by qPCR in samples obtained from pools of 20 larvae at 3 dpf and sharing the same mutation. Values are normalised to the expression levels of the *gapdh* gene and expressed as mean ± SD. *mecp2—methyl cpg binding protein 2*; *cdkl5—cyclin dependent kinase like 5*. Similar superscript letters indicate no statistically significant difference (*p* > 0.05). Different superscript letters indicate significant difference within column (*p* < 0.05).

To evaluate if zebrafish Mef2c loss of function affected brain development, the expression of *mecp2*, a crucial molecule for normal brain development [10], and of *cdkl5,* a critical regulator of neuronal morphogenesis [24], were analysed by qPCR, in homozygous single *mef2ca* mutants, single *mef2cb* mutants, double mutants and WT larvae. Expression of the *mecp2* gene was found to be decreased in all mutants analysed when compared to WT. The expression of the *cdkl5* gene was decreased in *mef2cb* homozygous single mutants and double-mutant larvae and maintained in *mef2ca* homozygous single mutants (Figure 7). These results indicate that Mef2ca and Mef2cb exert a positive regulation in the expression of the *mecp2* and *cdkl5* genes.

### 3.4. Early Motor Behaviour

**Figure 8:**
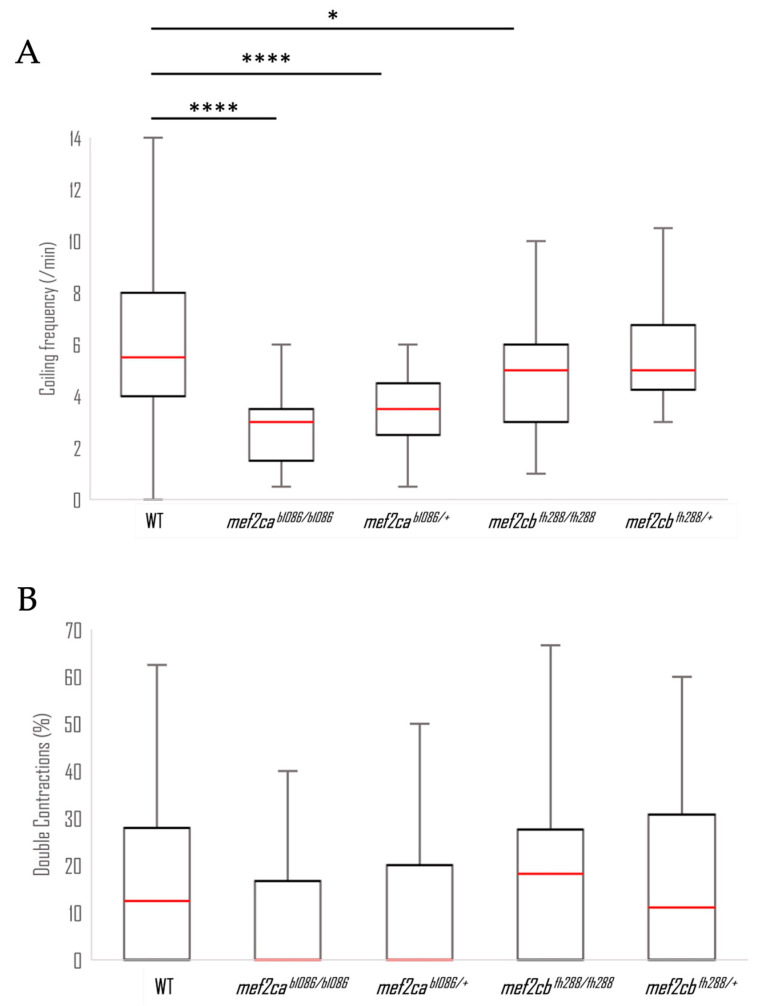
Comparison between wild-type and *mef2c* mutant embryos’ motor behaviour. (**A**) Frequency of coiling in wild-type (N = 46), *mef2ca^b1086/b1086^* (N = 19), *mef2ca^b1086/+^* (N = 46), *mef2cb^fh288/fh288^* (N = 53) and *mef2cb^fh288/+^* (N = 42) 25 hpf embryos, in a period of 2 min. (**B**) Double contraction percentage. The multiple contraction events were extracted from the video support for the five groups analysed. Results are presented as a box-and-whiskers graph. Significant statistical differences are indicated by * for *p* < 0.05 and **** for *p* < 0.0001.

The spontaneous contractions of wild-type, heterozygous and homozygous *mef2ca* and *mef2cb* mutant embryos of 25 hpf were monitored and quantified. Wild-type embryos presented a median frequency of coiling of 5.5 events per minute at 25 hpf (Figure 8A), significantly different from *mef2ca^b1086/b1086^* with 3 events per minute, *mef2ca^b1086/+^* with 3.5 events per minute and *mef2cb^fh288/fh288^* with 5 events per minute. *The mef2cb^fh288/+^* presented a median frequency of coiling of five events per minute. During the recording period, 12.5% of wild-type embryos exhibited at least one event with more than one contraction (Figure 8B). In *mef2ca^b1086/b1086^* and *mef2ca^b1086/+^*, none of the embryos showed events with more than one contraction. The *mef2cb^fh288/fh288^* and *mef2cb^fh288/+^* embryos showed at least one event with more than one contraction in 18.2% and 11.1% of the cases, respectively. None of the groups showed significant differences between them.

### 3.5. Spontaneous Swimming Activity

**Figure 9:**
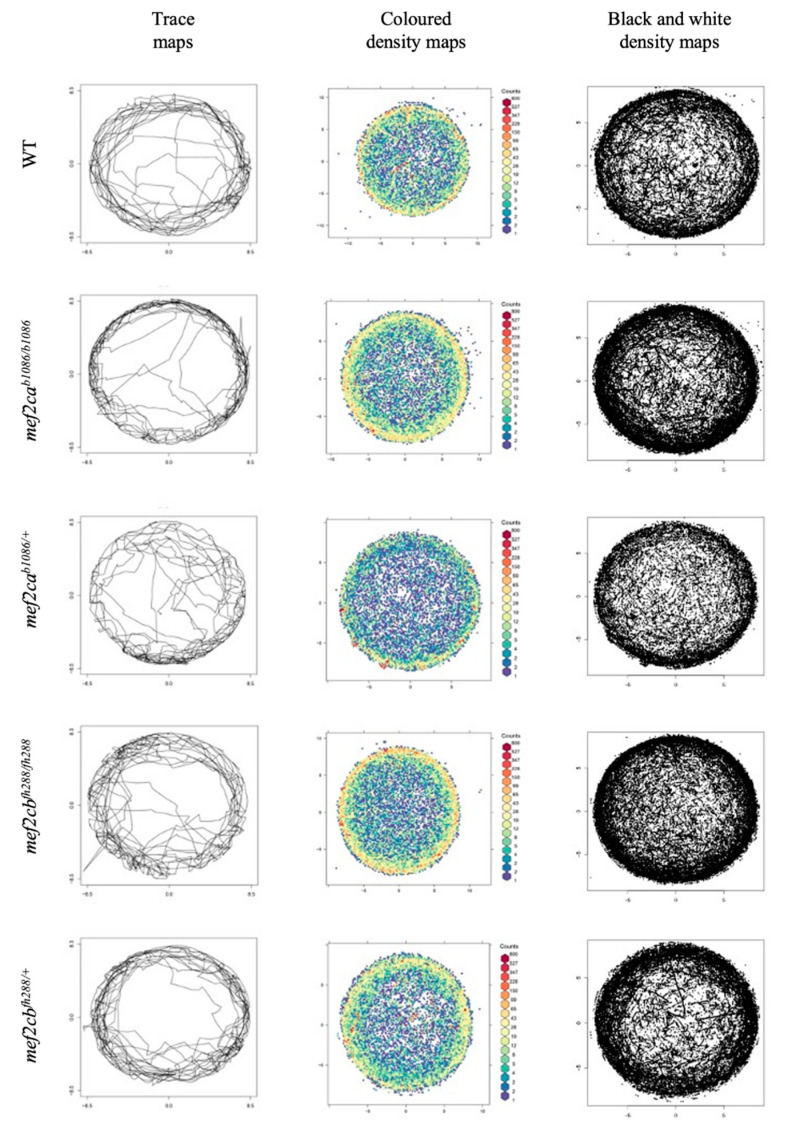
Trace and density maps. Example of traces for all genotype larvae, obtained from a recording session of 3 min (**left**). Coloured density maps represent the proportion of larvae at each position in the well (**middle**). Black and white density maps represent the proportion of larvae at each position in the well (**right**). Top to bottom: WT, *mef2ca^b1086/b1086^*, *mef2ca^b1086/+^*, *mef2cb^fh288/fh288^* and *mef2cb^fh288/+^*.

Differences between wild-type and *mef2ca* or *mef2cb* mutant fish were also assessed by monitoring their spontaneous swimming behaviour at 6 dpf, according to the methodology proposed by Pietri et al. (2013) [14]. To understand the overall positional behaviour of the fish in the arena over the 3 min time frame, we constructed maps of trace and density (the data agglomerate of each genotype) (Figure 9). The group of maps on the left represents an example of the trace path of one fish, while the other two maps represent the set of all the fish with the same genotype. Furthermore, the coloured map (middle) indicates the density (number of occurrences) in each position of the arena. Here, a preference for the borders of the well is visible either for wild-type, *mef2ca^b1086/b1086^*, *mef2ca^b1086/+^*, *mef2cb^fh288/fh288^* or *mef2cb^fh288/+^* larvae.

We performed histograms of the distance to the centre of the arena for the five genotype groups (Figure S1). An axis inversion was performed to facilitate distribution adjustment and visualization. The individuals tend to adopt different swimming patterns with the wild-type group keeping a preference for swimming along the wall of the arena, while the *mef2cb^fh288/+^* larvae showed a different circling behaviour along the dish compared with all the other groups, visible in the histogram.

**Figure 10:**
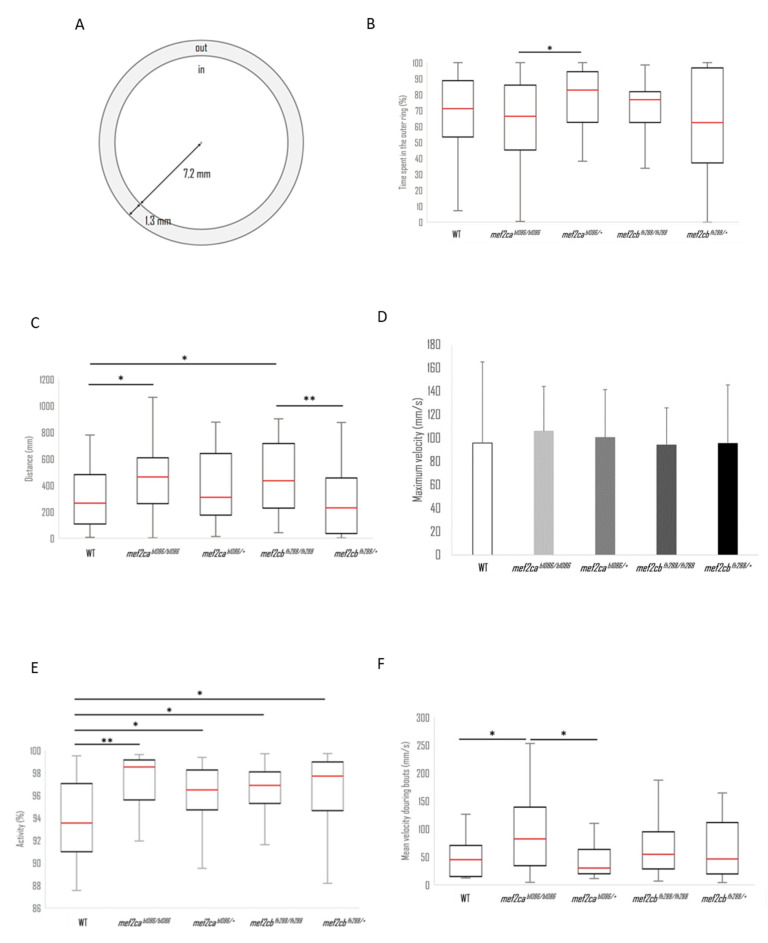
Analysis of trajectories for wild-type (N = 46), *mef2ca^b1086/b1086^* (N = 19), *mef2ca^b1086/+^* (N = 46), *mef2cb^fh288/fh288^* (N = 53) and *mef2cb^fh288/+^* (N = 42) larvae. (**A**) The arena-surface division into inner and outer regions used for data analysis. (**B**) Time percentage spent in an outside disk. (**C**) Total distance covered by each individual in a group during the recording time of 3 min. (**D**) Maximum velocity median of every group during the recording time of 3 min. (**E**) Mean velocity during movement bouts. (**F**) Median percentage of time in activity. Significant statistical differences are indicated by * for *p* < 0.05 and ** for *p* < 0.01.

Next, we analysed the thigmotactic responses of the different mutant larvae compared with WT. Thigmotactic behaviour describes the position of the larvae relative to the wall of the well, over time. The wells were divided into two zones: an inner circular zone at the centre of the well covering 72% of the total surface area and an outer ring region covering the remaining 28% (Figure 10A). Although there were visible differences between the groups, only the *mef2ca^b1086/b1086^* larvae, which spent 66.4% of the time in the outer ring, and *mef2ca^b1086/+^*, which spent 82.8%, presented a statistically significant difference (*p* < 0.05) (Figure 10B). It is interesting to note that *mef2cb^fh288/+^* presented a higher variability, with a larger inter-quartile distance (41.1% to 95.1%) in line with a flatter histogram (Figure S1), meaning that this group exhibits a higher tendency to swim along the entire radial distance. The median values of the swimming radial distance are summarized in Figure S2, with WT larvae swimming closest to the wall and *mef2cb^fh288/+^* larvae being those that swim more distant from the wall.

The total distance covered by each fish during the recording session of 3 min duration and the maximum velocity during that time were calculated. WT and *mef2cb^fh288/+^* larvae showed significantly shorter distances than the other groups (Figure 10C). The histograms of the distance covered by each of the five genotypes show that the distribution of the total distance covered by the WT larvae is different from all the other groups of larvae (Figure S3). The maximum velocity was similar for all the groups (Figure 10D).

To better understand the bouts of activity, we analysed the average acceleration of each group of fish as well as the maximum acceleration. Even though *mef2ca^b1086/+^* mutants have a lower median of the average acceleration, its box plot has a greater amplitude, greater than all the other groups, which may be indicative of quite erratic behaviour (results not shown). To further analyse the velocity pattern, the mean velocity during activity was calculated. Only velocities greater than 0.005 mm/s were considered to accommodate both the resting periods with zero velocity and drifting velocities due to medium flow or tracking errors. The results show that *mef2ca^b1086/b1086^* fish have a higher average velocity during movement than their wild-type counterpart which is confirmed by the Kruskal–Wallis test, with a level of confidence lower than 5% (Figure 10E). Interestingly, the homozygous mutant for the *mef2ca* mutation exhibits differences from the heterozygous mutant, with a confidence level of 5%, according to the Kruskal–Wallis significance test. The two mutant fish for the *mef2cb* gene, homozygous and heterozygous mutants, presented an average velocity during movement not significantly different from the WT larvae or each other (Figure 10E).

Next, we determined the percentage of time that larvae spent swimming (Figure 10F). The WT larvae showed an activity of 93.5% (quartile percentage times ranging from 91 to 97%) significantly different from *mef2ca^b1086/b1086^* mutant larvae with 98.5%, *mef2ca^b1086/+^* with 96.5%, *mef2cb^fh288/fh288^* with 96.9% and *mef2cb^fh288/+^* with 97.7% of the time spent swimming.

Overall, these results suggest that *mef2c* mutant larvae have impaired locomotion and increased thigmotaxis.

## 4. Discussion

The zebrafish *mef2ca^b1086^* and *mef2cb^fh288^* mutant lines, known as loss-of-function lines, were described in previous works focusing on the craniofacial and cardiac phenotypes [7,8]. The cardiac phenotype for the homozygous double mutants was characterized showing that Mef2ca and Mef2cb function redundantly and are essential to driving cardiomyocyte differentiation [8]. The craniofacial phenotype was only described for larvae homozygous for *mef2ca^b1086^* and heterozygous for *mef2cb^fh288^*, showing an increased disarrangement observed in the cartilaginous structures when compared to homozygous larvae for the *mef2ca^b1086^* mutation. Homozygous larvae for the *mef2cb^fh288^* mutation showed a phenotype indistinguishable from that of WT larvae [8]. DeLaurier and colleagues (2014) [9] suggested that Mef2ca and Mef2cb have partially redundant functions in cartilage development but not in bone development. Mutant lines, in which the mutation provides a null allele, represent a functional tool to further investigate protein functions and their molecular interactions.

The severe cardiac phenotype observed in homozygous double-mutant zebrafish larvae, which caused the death of the specimens by 6 dpf because of the disruption of the heart due to fluid accumulation inside the cardiac pericardium, complements the phenotype previously described by Hinits and colleagues (2012) [8]. These results are in agreement with those observed in mice, where a knockout of *Mef2c* led to the disruption at day 9 of embryonic development due to anomalies occurring in heart development [25]. Our results confirm the crucial role of Mef2ca and Mef2cb in zebrafish heart development, providing evidence for the maintenance of this function throughout evolution. Furthermore, because of the crucial role of *Mef2c* in cardiac development, the use of mice as a model for its inherent diseases is hampered because they die during embryonic development [25]. The use of zebrafish represents an alternative for further investigation of the pathways affected by Mef2c.

We have also further analysed the defects associated with craniofacial cartilage and bone development in the homozygous double mutants. The alcian-blue-stained cartilage structures allowed us to describe, for the first time, the craniofacial cartilaginous phenotype associated with the absence of both MEF2C co-orthologues in zebrafish. A profound disarrangement of cartilaginous structures was observed, resulting in a smaller size and deformed structures, originating a non-functional mouth. The craniofacial bone phenotype of the homozygous double-mutant larvae was analysed using the alizarin red S live staining technique. We observed phenotypic alterations of the ethmoid plate, opercula, otoliths, basioccipital process and pharyngeal teeth. Our study expands and complements data previously reported by other authors [7,9], providing additional insights towards understanding the effects of Mef2c loss of function and in particular evidencing that in the absence of both Mef2ca and Mef2cb, zebrafish shows severe craniofacial defects.

The two transgenic mutant lines Tg(*-4725sox10:GFP*);*mef2ca^b1086/^*^b1086^ and Tg(*-4725sox10:GFP*);*mef2ca^b1086/b1086^*;*mef2cb^fh288/fh288^* developed in this study carry the *mef2ca^b1086^* and *mef2ca^b1086^;mef2cb^fh288^* mutations, respectively, and express GFP under the control of *sox10* promoter. Therefore, these lines allow in vivo imaging of the cartilaginous craniofacial phenotypes developed, since *sox10* is expressed in cranial neural crest (CNC) cells that will give rise to craniofacial and neuronal structures as previously validated [17]. The use of these reporter lines allowed us to follow the development of the craniofacial phenotype associated with larvae homozygous for *mef2ca^b1086^* mutation until 10 dpf and homozygous double-mutant larvae *mef2ca^b1086^*;*mef2cb^fh288^* until 6 dpf, clearly showing the profound disarrangement of the craniofacial cartilaginous structures from these mutant lines, already demonstrated by us using the alcian blue staining technique, but allowing us to follow the same individuals throughout time.

Since these transgenic lines mark also neuronal cells such as the interneurons and oligodendrocytes [17], they are also a valuable tool for further analysis concerning the disclosure of the neuronal phenotype of these mutant lines. In addition, these lines represent an important in vivo tool to assess the effect of potential therapeutic drugs capable of rescuing the severe phenotype caused by these mutations and could be used as a first line of screening before mammalian testing.

To determine if the altered expression of *MECP2* and *CDKL5*, observed in patients with MEF2C haploinsufficiency syndrome [12], also occurs in the zebrafish mutants for *mef2ca* and/or *mef2cb*, we determined *mecp2* and *cdkl5* expression levels and found a significant decrease in *mecp2* expression, in *mef2ca* and *mef2cb* single mutants, but no further effect was observed in the double mutants, arguing against a synergistic effect of the two orthologues. In contrast, *cdkl5* expression was decreased in *mef2ca* and *mef2cb* single mutants, and this effect was further incremented in the double mutants suggesting a synergistic effect between both co-orthologues. Our results indicate that both co-orthologues of *MEF2C* affect, in a positive manner, the regulation of *mecp2* and *cdkl5* genes, providing the first insight towards *cdkl5* function in zebrafish and showing that Mef2c regulation of *cdkl5* and *mecp2* is conserved in zebrafish, as it was already shown to occur in humans [12]. These results provide the first description of neuronal development marker deregulation in zebrafish *mef2c* mutants. The link between the mutant phenotype and the dysregulation of the marker genes needs to be further investigated, and more genes should be analysed to better characterize the observed phenotypes and relate them to genotypes, for instance, by performing RNA-seq.

In this study, we also performed a kinematic analysis of the motor activity in embryos and young *mef2ca* or *mef2cb* mutant larvae. Our results reveal alterations in spontaneous motor behaviours. In zebrafish, spinal motor neurons are known to make synapses on the axial muscles, and the adequate neuronal morphogenesis of motor neurons is critical for achieving context-specific synaptic drive at the level of muscles, enabling the organism to execute motor behaviours [26].

We have tested the zebrafish *mef2c* mutants for motor dysfunctions in 6 dpf larvae in the open-field paradigm. We observed that alteration of Mef2c functions in zebrafish larvae induced an increase in the activity level, notably characterized by an increase in the total distance covered and the time spent swimming. Interestingly, the mean velocity during movement bouts was higher in *mef2ca^b1086/b1086^* mutant larvae compared with the wild-type larvae. We may hypothesize that suppression of Mef2c functions in zebrafish larvae, particularly *mef2ca* expression, may induce disruption of the neuromodulation of the motor network development. We have also tested for thigmotaxis in 6 dpf larvae. In thigmotaxis, an animal avoids the centre of an arena and moves towards the edge or periphery of a novel environment such as a wall [27]. Zebrafish larvae with thigmotaxis prefer to stay near the wall of the wells [28]. Our results showed that *mef2c* mutant larvae spent the same time close to the well’s edge as wild-type larvae. This may suggest that mutant larvae do not experience deregulation of anxiety behaviours. However, the mutant larvae covered more total distance than their WT counterparts, a variable that has also been shown to be indicative of anxiety levels in mice [29].

In summary, we described the craniofacial phenotype of the dual loss of expression of *MEF2C* orthologues in zebrafish, and we demonstrated that both have a function in cartilage, bone and brain development. We also developed mutant transgenic reporter lines with *mef2ca^b1086^* and *mef2cb^fh288^* mutations, which can be used for further studies concerning phenotypic reversions. In addition to the conventional choice of rodents as models to perform studies concerning genetic alterations in diverse human diseases, zebrafish has become progressively considered an important complementary model for translational research, as it consists of the only suitable vertebrate model for large-scale throughput drug screening. The analysis of *mef2ca* and *mef2cb* null zebrafish during early development did uncover craniofacial defects and molecular deregulation compatible with MEF2C-null mouse models and MRD20 patients. Altogether, our results highlight zebrafish *mef2c* mutants as valuable models for future studies concerning MEF2C-related pathologies. Furthermore, information on the regulation of MeCP2 and CDKL5 will also be invaluable towards understanding their molecular interactions with MEF2C both in health and in disease stages.

## Figures and Tables

**Table 1:** List of primers used in this study.

Gene	Accession ID	Sequence 5′-3′
methyl CpG binding protein 2 (*mecp2*)	AY298900.2	Fw: AGAGACCTTTGAGAAACGACTG Rev: TCTTCTTGTGACTCTTCGGTG
Cyclin-dependent kinase-like 5 (*cdkl5*)	NM_001145768.1	Fw: AGATGAACCGAAGCCTACTGA Rev: GGTGTATCCAAGACCGTAA
glyceraldehyde 3 phosphate dehydrogenase (*gapdh*)	NM_001115114.1	Fw: GCCCACCAGAACATCATCCCTGC Rev: TGACAGACTCCTTGATGTTGGCGTAG

## Data Availability

The data presented in this study are available in Supplementary Material here.

## Supplementary Materials

The following supporting information can be downloaded at https://www.mdpi.com/article/10.3390/biom13050805/s1, Figure S1: Histograms of the radial distance from the wall for the five groups analysed: WT (*N* = 26), *mef2ca^b1086/b1086^* (*N* = 29), *mef2ca^b1086/+^* (*N* = 19), *mef2cb^fh288/fh288^* (*N* = 30) and *mef2cb^fh288/+^* (*N* = 30). Notice the inversion of the axis direction to facilitate distribution adjustment; Figure S2: Location of the median of the swimming radial distance for the five groups analysed: WT (*N* = 26) in blue, *mef2ca^b1086/b1086^* (*N* = 29) in red, *mef2ca^b1086/+^* (*N* = 19) in yellow, *mef2cb^fh288/fh288^* (*N* = 30) in green and *mef2cb^fh288/+^* (*N* = 30) in orange; Figure S3: Histograms of the total distance covered by each fish for the five groups analysed: WT (*N* = 26), *mef2ca^b1086/b1086^* (*N* = 29), *mef2ca^b1086/+^* (*N* = 19), *mef2cb^fh288/fh288^* (*N* = 30) and *mef2cb^fh288/+^* (*N* = 30).
