# Supplementary material for: mef2ca and mef2cb Double Mutant Zebrafish Show Altered Craniofacial Phenotype and Motor Behaviour
Source: Biomolecules. 2023 May 9;13(5):805. doi: 10.3390/biom13050805 (PMC10216501; doi:10.3390/biom13050805)
Supplement: Supplementary file 1 [file biomolecules-13-00805-s001.zip › biomolecules-2341274-supplementary.pdf]

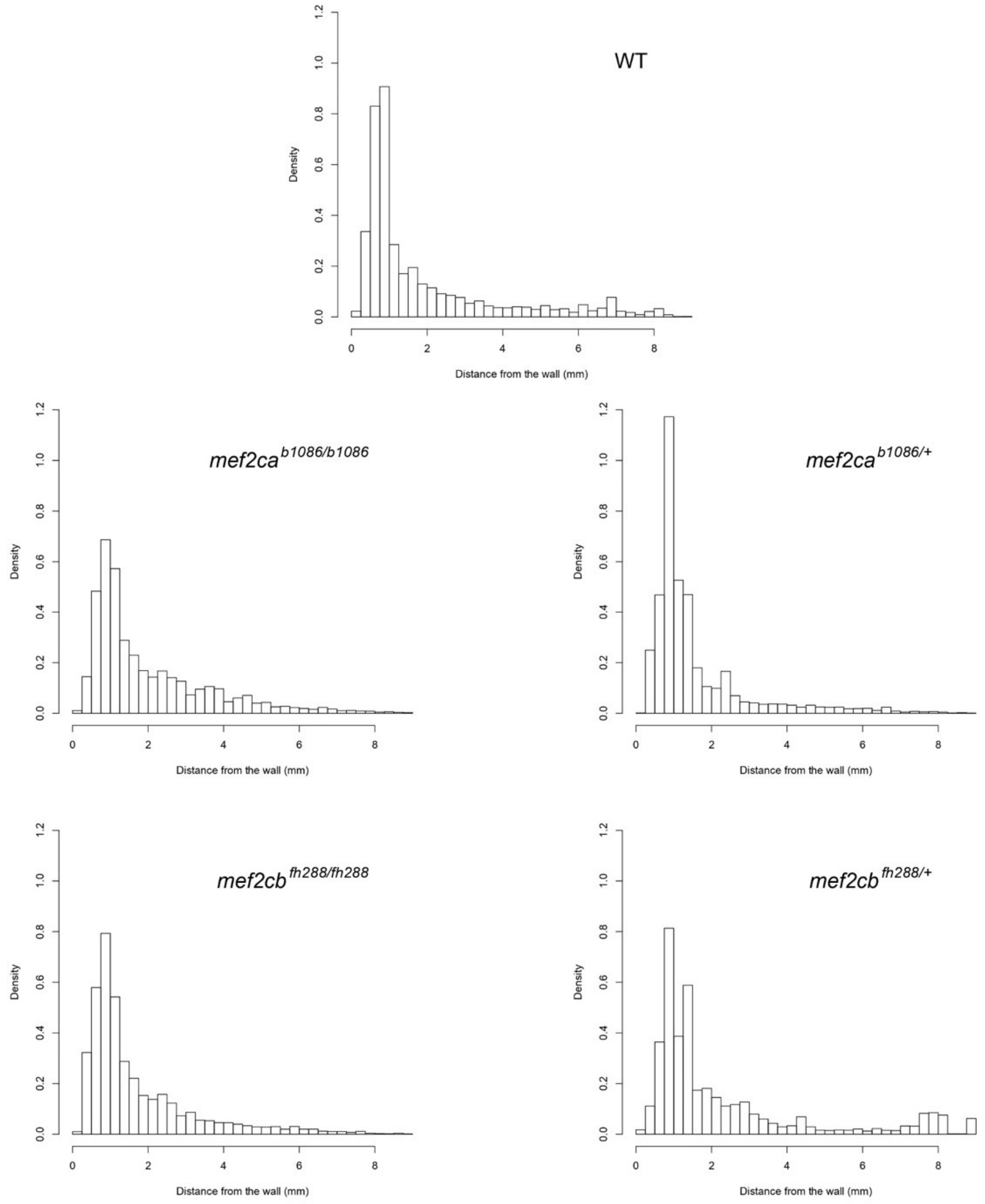

Figure S1: Histograms of the radial distance from the wall for the five groups analysed: WT (N=26), *mef2ca*$^{b1086/b1086}$ (N=29), *mef2ca*$^{b1086/+}$ (N=19), *mef2cb*$^{fh288/fh288}$ (N=30) and *mef2cb*$^{fh288/+}$ (N=30). Notice the inversion of the axis direction to facilitate distribution adjustment.

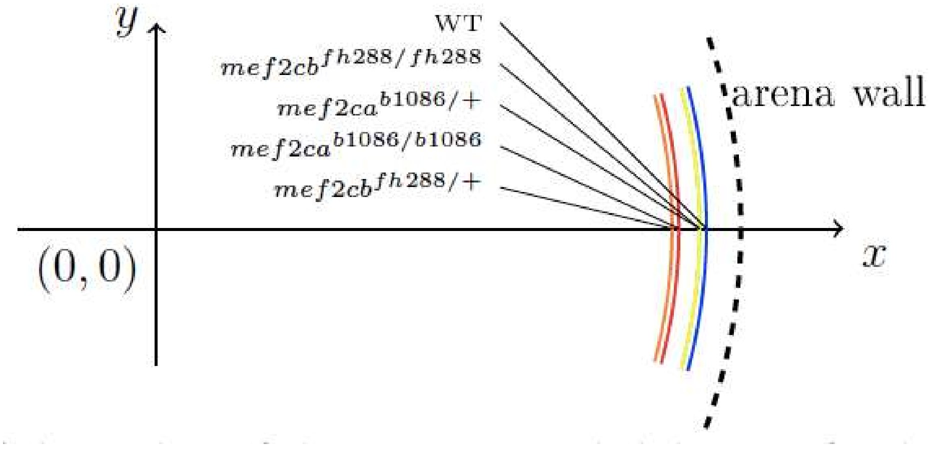

Figure S2: Location of the median of the swimming radial distance for the five groups analysed: WT (N=26) in blue, *mef2ca*$^{b1086/b1086}$ (N=29) in red, *mef2ca*$^{b1086/+}$ (N=19) in yellow, *mef2cb*$^{fh288/fh288}$ (N=30) in green and *mef2cb*$^{fh288/+}$ (N=30) in orange.

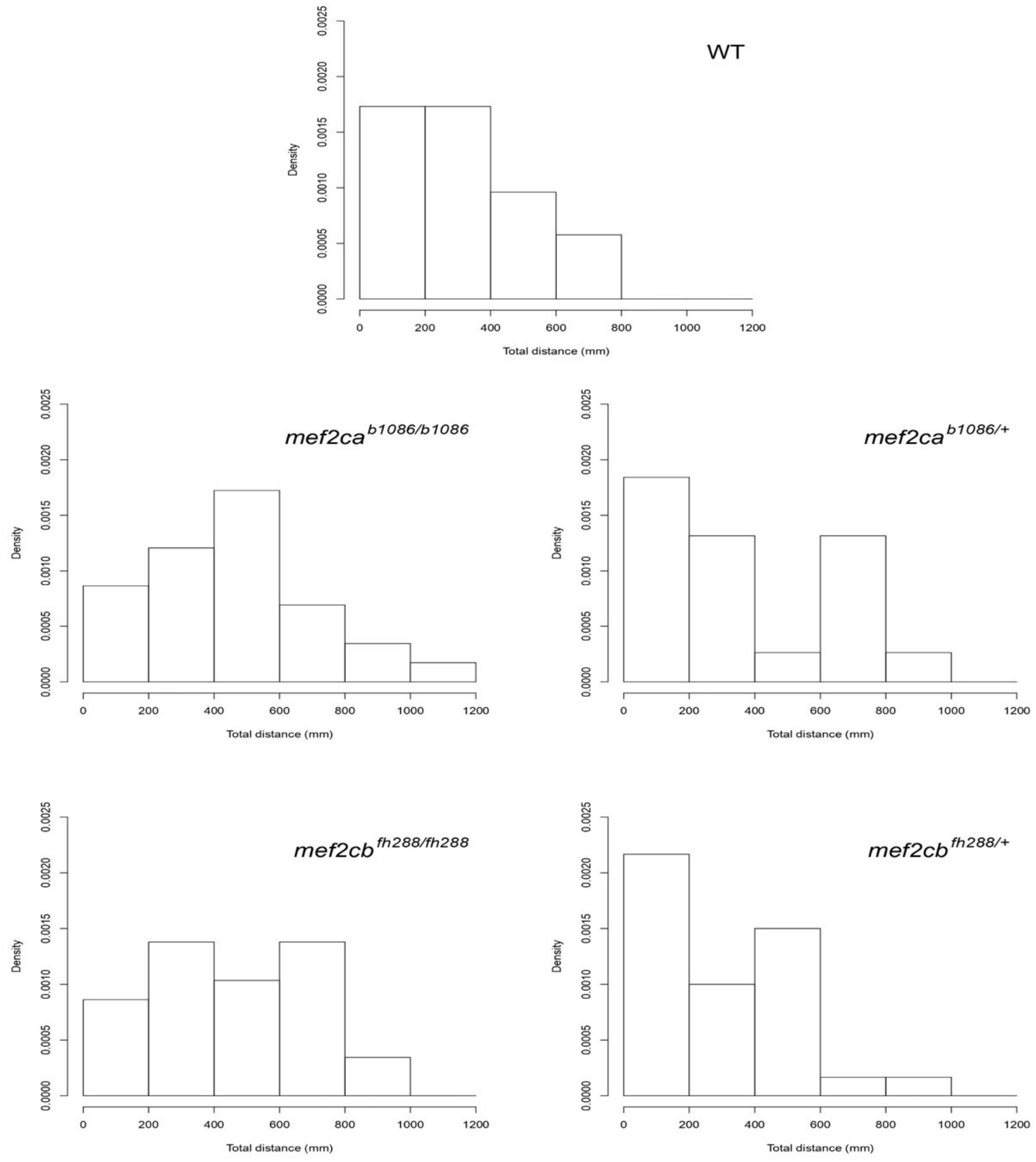

Figure S3: Histograms of the total distance covered by each fish for the five groups analysed: WT (N=26), $mef2ca^{b1086/b1086}$ (N=29), $mef2ca^{b1086/+}$ (N=19), $mef2cb^{fh288/fh288}$ (N=30) and $mef2cb^{fh288/+}$ (N=30).
